# Visual Haptic Feedback for Training of Robotic Suturing

**DOI:** 10.3389/frobt.2022.800232

**Published:** 2022-02-02

**Authors:** François Jourdes, Brice Valentin, Jérémie Allard, Christian Duriez, Barbara Seeliger

**Affiliations:** ^1^ InSimo SAS, Strasbourg, France; ^2^ DEFROST Team, UMR 9189 CRIStAL, CNRS, Centrale Lille, Inria, University of Lille, Lille, France; ^3^ IHU-Strasbourg, Institute of Image-Guided Surgery, Strasbourg, France; ^4^ Department of General, Digestive, and Endocrine Surgery, University Hospitals of Strasbourg, Strasbourg, France; ^5^ ICube, UMR 7357 CNRS, University of Strasbourg, Strasbourg, France; ^6^ IRCAD, Research Institute Against Digestive Cancer, Strasbourg, France

**Keywords:** surgical simulation, haptics, knot-tying, collision detection, virtual reality, robotic surgery training, minimally invasive surgery

## Abstract

Current surgical robotic systems are teleoperated and do not have force feedback. Considerable practice is required to learn how to use visual input such as tissue deformation upon contact as a substitute for tactile sense. Thus, unnecessarily high forces are observed in novices, prior to specific robotic training, and visual force feedback studies demonstrated reduction of applied forces. Simulation exercises with realistic suturing tasks can provide training outside the operating room. This paper presents contributions to realistic interactive suture simulation for training of suturing and knot-tying tasks commonly used in robotically-assisted surgery. To improve the realism of the simulation, we developed a global coordinate wire model with a new constraint development for the elongation. We demonstrated that a continuous modeling of the contacts avoids instabilities during knot tightening. Visual cues are additionally provided, based on the computation of mechanical forces or constraints, to support learning how to dose the forces. The results are integrated into a powerful system-agnostic simulator, and the comparison with equivalent tasks performed with the da Vinci Xi system confirms its realism.

## 1 Introduction

Traditionally, in open surgery, surgeons’ fingertips sensed tension on tissue and suture threads. In minimally invasive surgery, sensory perception is reduced as the interaction is mediated by an instrument. In robot-assisted surgery, tele-manipulation implies that there is no more direct physical interaction between surgeon and patient. Force sensing and feedback could transmit a sense of touch, but they are not typically available in current platforms Amirabdollahian et al. (2018); Miller et al. (2021). Consequently, surgeons rely on visual cues such as tissue distensibility and deformation, in order to get a “feel” for it. Perception of visual input as a tactile sense is an acquired skill and needs considerable practice.

Suturing and knot tying are basic surgical skills required for various procedures amongst all surgical specialties. In minimally invasive surgery, sutures are performed with long and rigid instruments in angles determined by the trocar positions. These constraints have to be overcome by optimal needle and instrument positioning and by a sometimes challenging surgical technique. Robotic surgical systems provide an enhanced interface with articulating instruments reproducing surgeons’ wrist movements and angulation. However, robotic control interfaces typically do not provide force or tactile feedback. Thus, the application of excessive force is a common phenomenon when robotic novices familiarize with a system. During suturing tasks, such shearing and tearing forces lead to needle deformation or breaking as well as suture breakage. Training on the robotic system is needed to adapt the suturing technique in order to apply the correct amount of tension for tightening a suture or knot without too much strain. In the recent systematic review of Golahmadi et al. (2021), assessing instrument-tissue interaction forces exerted during surgery across different specialities, the comparison of mean average forces between groups showed that novices exerted 22.7% more force than experts, and that the presence of a feedback mechanism reduced exerted forces by 47.9%. Thus, trainees particularly benefit from force feedback.

Suture breakage is significantly more common in surgeons inexperienced in robotics when compared to trained robotic surgeons. Reiley et al. (2008) demonstrated that visual force feedback (VFF) is particularly advantageous for novice surgeons, with significantly reduced suture breakage rates, as well as peak and standard deviations of applied forces during knot-tying with a da Vinci robotic system equipped with force-sensing instrument tips. In contrast, the presence or absence of VFF did not change measured performance parameters among experienced robotic surgeons. It was suggested that VFF is better than physical force feedback in terms of knot quality. Interestingly, visual haptics also significantly reduced information flow as determined by brain wave sensing, suggesting a lower cognitive burden with superimposed visual cues during robot manipulation in a VR environment, while increasing task performance as presented by Haruna et al. (2020).

Virtual Reality (VR) simulators provide a unique training environment with an unlimited number of repetitions possible for suturing and knot tying tasks and practice. Additional visual cues can be integrated to enable VFF. A suture training model for robotic surgery requires the integration of a realistic suture thread performance in the virtual simulator environment. The present work proposes a new simulation approach for suture training, based on precise mechanics of suture behavior and its interactions with tissues. It integrates a realistic thread simulation for suturing and knot-tying, as well as a real-time computation of the stress fields, especially on the thread. A colour-coded force feedback based on the stress fields can be activated to alert surgeons when excessive force is applied during VR robotic suturing, in order to avoid excessive shearing forces. These haptic cues illustrating the influence of trainees’ gestures on the mechanical balance can enhance training to adapt to the absence of tactile feedback in the non-virtual robotic surgery environment.

To build such a simulator, the best possible trade-off between physics realism and latency has to be found. Latency to compute physics has to be compatible with visual rendering (minimum 30 Hz) and haptic rendering (500 Hz to 1 kHz). At the same time, latency has to be minimized since it decreases surgical performance, as shown in robotic long-distance telesurgery studies like Marescaux et al. (2001) and Doarn et al. (2009). If fast (latency-less) simulation comes at the price of model inaccuracy in reproducing knot-tying or sutures, learning of the entire sequence is impaired. Therefore, a number of technical challenges have been answered in this study with new methods that are presented in this paper. Our approach is based on the work of Guébert et al. (2009) to which we bring the following contributions:

• The mechanical model of the suture thread, based on the theory of beams in global coordinates is constrained in length. The formulation is compatible with the calculation of mechanical stress on the thread and allows for adding visual cues and simulation (detection) of breakage,
• A smooth collision and contact response formulation based on curves allows for more consistent behavior than with serial segments,
• The real-time simulation of knot tying in a stable and physical way is simulated up to the end of the tightening,
• A realistic suture training simulator is demonstrated on basic exercises, where they can be compared to videos of training actually performed with a surgical robot.

## 2 Related Work

### 2.1 Virtual Reality Robotic Surgery Simulators

#### 2.1.1 Commercial Products

There are two different types of robotic surgery VR simulators: the specific “backpack” ones for each of the commercial platforms (e.g., *SimNow*, Intuitive Surgical, United states) and three stand-alone systems (*dV-Trainer*, Mimic Technologies, United states; *RobotiX Mentor*, 3D Systems/Simbionix, United states; *Robotic Surgery Simulator*, Simulated Surgical Systems, United states), of which both Simbionix and Mimic Technologies are now part of Surgical Science Sweden AB.

Due to the challenges to realistically model suture threads and their behavior in simulation environments, several “cheats” are commonly used throughout these systems to provide speed and stability in the simulation modules. As an example, suture threads not respecting the property of inextensibility, or tools blocking when a certain tension is applied, prevent the trainee to learn how much a thread can be stretched before breakage. Moreover, damped suture movements and unrealistic gravity forces lead to unnatural effects during knot-tying. Furthermore, automatic tightening of the knot when traction is applied on both ends of the thread impedes an adjustment of the knot position. Such adjustments are usually performed by adapting the traction in order to slide the knot towards one region of the thread to optimize the knot position and the length of both ends of the thread. The reason why such unrealistic behaviors are implemented is that they facilitate a fast and robust simulation. At the cost of missing technical details, users can thereby complete a given task while avoiding instabilities.

In contrast, we chose to model the suture with precise mechanical properties for a realistic training experience including suturing errors such as suture breakage, air knots and tissue tearing. However, a system of visual cues is implemented to alert the trainee of improper technique prior to reaching a “game over”.

#### 2.1.2 Haptic Rendering

Haptic rendering on surgical simulations has been the subject of numerous studies like Basdogan et al. (2004) and Marshall et al. (2006). There are many difficulties to achieve a quality haptic rendering (see the study of Hamza-Lup et al. (2019) for more information): A first challenge is to have a realistic modeling of surgical tools, of the mechanical behavior of the organs, as well as of the interaction between tools and organs. A second difficulty is based on the performance required for a realistic haptic rendering. In general, it is estimated that to simulate a haptic rendering of contact, the control must be performed at 1 kHz. Combining these two challenges already means that we need a very fast and very realistic simulation engine at the same time. But a third difficulty comes from the coupling between this simulation engine and the haptic rendering. Indeed, the robotic interface requires a control law that guarantees stability. But this control law will often disturb the quality of the haptic rendering (called its transparency).

In the end, these difficulties mean that the haptic rendering is often disappointing on surgical training simulators, and does not necessarily provide educational value, as reported by Rangarajan et al. (2020). In our study we therefore choose to only provide visual cues translating this force feedback, even if our approach theoretically allows for haptics. It should be noted that most surgical robots (notably the Da Vinci) do not offer force feedback, so it is logical that the corresponding simulator does not provide any either.

### 2.2 Deformation Model for the Suture Thread

We identified two main properties which need to be captured to faithfully represent the deformation of a suture thread. The first one is the ability to form loops and knots, which stems from the mechanical coupling between bending and torsional motion. The second one is the conservation of the length, which adds visual realism, and allows to perform accurate tying motions. Different families of methods exist for modeling the deformation of one-dimensional structures, also denoted as rods, ranging from purely geometric models (Brown et al., 2004; Müller et al., 2012), to more elaborate ones which derive from either Euler Bernoulli, Timoschenko, Kirchhoff or Cosserat theory. Essentially, the more complex the theory, the richer is the deformation of the model. This section introduces previous work on deformation models used to model elastic rods, categorized into two families: reduced coordinate methods, and maximal coordinate methods.

#### 2.2.1 Reduced Coordinate Methods

With reduced coordinate methods, the number of parameters to describe the motion exactly matches the degrees of freedom of the model. As a consequence, the inextensibility property is captured natively by the model. In this setting the Cartesian coordinates of the rod centerline are an implicit function of the degrees of freedom. A tempting approach is to consider the suture thread as a particular case of a chain of articulated rigid segments, where the angles between two adjacent segments are the degrees of freedom. Using the method described by Featherstone (1983), this type of model can be solved in linear time. Rigid articulated chains perfectly preserve length, but have the major drawback of not capturing torsional motion, which plays a critical role for the formation of knots.

Bertails et al. (2006) derived a model using Kirchhoff theory, where the curvature is used as the degree of freedom of the rod. Very complex deformations can be obtained even with a coarse discretization, and both torsional and bending motions are captured by the model. A recursive solving strategy, similar in spirit as the one introduced by Roy Featherstone for articulated rigid chains, can be applied to recover the rod Cartesian coordinates from the curvature degrees of freedom in linear time (Bertails, 2009).

However, even though reduced coordinates offer an attractive formulation to solve the suture thread deformation, they require additional implementation work in order to be integrated efficiently in a simulation environment which contains other types of deformable solids. To scale well, reduced coordinates need a dedicated linear solver to alleviate for the need explicit assembly of the system matrix. Indeed the system dynamic matrix is always dense, and therefore the complexity of a direct solve is cubic with the number of degrees of freedom. Also, due to the implicit nature of the formulation, the expression of the rod centerline position and velocity can only be recovered with the application of an additional geometric function.

#### 2.2.2 Maximal Coordinate Methods

With maximal coordinate methods, the number of parameters used to describe the model is greater than the degrees of freedom, so additional constraints are needed, e.g., to preserve the length of the model. On the numerical side, the system matrix is very sparse, so the application of its inverse can be computed efficiently using linear algebra routines like the conjugate gradient method (Shewchuk, 1994) or the direct sparse Cholesky factorization (Davis, 2006).

Mass-spring models are probably the simpler conceptual implementation for elastic rods, but they lack mechanical soundness. The rod centerline is discretized with a succession of segments, connected by linear springs. Choe et al. (2005) also added torsion to this type of model by tracking the difference in orientation between two successive segments. Spline based models by Lenoir et al. (2004) and Theetten et al. (2006) offer a continuous representation of the rod centerline, and torsion is obtained by tracking the change in orientation of the rod cross section.

Work by Spillmann and Teschner (2007) introduced the simulation of elastic rods to the computer graphics community by discretizing the Cosserat rod theory. In this setting the degrees of freedom are described by a spatially continuous material frame, called the Frenet frame, which is computed by taking the parametric derivatives of the rod centerline curve, and allows to measure both bending and torsion on top of stretching. This work was later extended by Bergou et al. (2008) who proposed a different framing for the material basis, called the Bishop framing. Contrary to Frenet framing, Bishop framing is always defined even in regions where the curvature of the centerline is zero. In Bergou et al. (2010), they provided the second derivative of the energy function of this model which makes it suitable for implicit time integration with large time steps (Baraff and Witkin, 1998).

Guébert et al. (2009) proposed to model a suture thread by resorting to the Timoschenko beam theory (Przemieniecki, 1985), which is traditionally used for the analysis of structures, and equipped it with a corotational filter to account for large displacements.

Among the family of maximal coordinates method, a popular alternative to implicit time integration is to use the Position Based Dynamics (PBD) framework (Müller et al., 2007; Macklin et al., 2016). Using just the first derivative of the energy function and a non-linear Gauss-Seidel solver, this method approximates implicit Euler integration. Position-based solving method is very robust, but requires a lot of iterations to converge to a stiff behavior. Work by Umetani et al. (2015) has shown how Cosserat rod theory can be expressed in the PBD framework using ghost particles to model orientations. Later this was work was extended by Kugelstadt and Schömer (2016) to treat orientation constraints directly in the PBD framework. However as demonstrated by Deul et al. (2018), and Xu and Liu (2018) in the more particular context of suture thread simulation, a large number of iterations are required to achieve stiff behaviors with Position-based methods, and propose to use a direct solver to obtain inextensible rods.

Still, since they allow stretching to occur, all the maximal coordinate formulations need an additional Lagrangian hard constraint in order to project the rod kinematics to a subspace where stretching motion is cancelled.

## 3 Mechanical Modeling

We present a novel simulation model of suture threads which employs:

• Corotational Timoshenko beam elements. This model accurately captures the motions of the thread and can be used to represent a large variety of suture threads ranging from very stiff to very soft.
• A discrete continuous geometrical model for the centerline based on cubic Bézier curves whose control points can be inferred from the beam configuration.
• A stable length-preserving second order Lagrange multiplier constraint based on the derivatives of the cubic Bézier curve length with respect to the beam generalized coordinates.
• A spatially continuous contact geometry which relies on the cubic Bézier curve geometry of the thread. It allows to produce an accurate and smooth contact detection which is particularly helpful during knot formation where the model becomes more and more tangled.

This section presents the parts of the overall simulation model that are specific to the suture thread. It will be illustrated with results presented in Section 4 within a full simulation including other rigid and soft bodies, linear solvers and constraints resolution schemes from the SOFA open-source software Faure et al. (2012), with additional methods and proprietary implementation from InSimo that improve the achieved performances, stability, and precision of the tissues modeling. However, the presented suture thread model is generic and standalone and could be used within other engines.

### 3.1 Equations of Motion

Using Newton’s second law, the acceleration of the objects can be related with the applied forces

$$\begin{array}{cc} M\ddot{q} & =f_{ext}-f_{int}+H^{⊤}\lambda \\ \Phi \left(q\right) & =0 \end{array}$$ (1)

Where $M\in R^{n\times n}$ is the mass matrix of the system, $q\in R^{n}$ are the generalized coordinates, **f**_*ext*_ and **f**_*int*_ are the external and internal forces, respectively. **H** ^*⊤*^ *λ* are the constraint forces, where $H\in R^{m\times n}$ is the Jacobian matrix gives the constraint force directions of the *m* algebraic constraint equations Φ, and $\lambda \in R^{m\times 1}\in$ is the vector of unknown Lagrange multipliers encoding constraint force intensities.

This equation is integrated using the backward Euler scheme to accommodate for large time steps, which was popularized by Baraff and Witkin (1998) in computer graphics. The system can be written in matrix form

$$\left(\begin{array}{cc} M-h^{2}K & -H^{⊤}\\ H & \frac{1}{h^{2}}C \end{array}\right)\left(\begin{array}{c} {\dot{q}}_{+}\\ h\lambda_{+} \end{array}\right)=\left(\begin{array}{c} M\dot{q}+h\left(f_{ext}-f_{int}\right)\\ -\frac{1}{h}\Phi \left(q\right) \end{array}\right)$$ (2)

With **K** the stiffness matrix coming from the linearization of the internal forces, **C** the optional compliance of the constraints, and *h* the time step. The subscript + denotes an end of time step quantity. This integration scheme allows to obtain an equation of the dynamics in velocity, with impulses in the right term. It is a low order scheme but well adapted to a “time stepping” processing of the contacts.

The reduced system can be formed by taking the Schur complement of the upper left block of the system matrix. Using **A** = **M** − *h* ^2^ **K** we obtain

$$\left(HA^{-1}H^{⊤}+\frac{1}{h^{2}}C\right)h\lambda_{+}=-\frac{1}{h}\Phi -HA^{-1}\left(M\dot{q}+h\left(f_{ext}-f_{int}\right)\right)$$ (3)

The symmetric semi positive definite matrix **W** = **HA** ^−1^ **H** ^*⊤*^ is the Delassus operator (Delassus 1917), or compliance matrix projected in the constraint space. It encodes the mechanical coupling between each of the constraint equations of the system.

To solve the system 2) we first compute the free velocity, which is the velocity obtained by removing the influence of the constraint forces

$${\dot{q}}_{*}=A^{-1}\left(M\dot{q}+h\left(f_{ext}-f_{int}\right)\right)$$ (4)

Then we compute the intensities of the constraint forces *λ*_+_ using the reduced system. This system usually models a mixed non linear complementary problem. It is called “mixed” since it combines both equalities and inequalities constraint equations, and constraint equations may be also be non linear, like with frictional contact constraints.

Finally once the intensities *λ*_+_ are obtained we can compute the end of time step velocity and position

$$\begin{array}{cc} {\dot{q}}_{+} & ={\dot{q}}_{*}+A^{-1}H^{⊤}\lambda_{+}\\ q_{+} & =q+h{\dot{q}}_{+} \end{array}$$ (5)

### 3.2 Deformation Model of the Suture Thread

To model the suture thread deformation we extend the Timoschenko linear beam finite element method presented in Przemieniecki (1985) to account for large displacements by using a corotational formulation.

#### 3.2.1 Corotational Beam Elements

We will provide a succinct introduction to the corotational method in this section, and we refer the reader to Felippa and Haugen (2005) for extensive details.

The generalized coordinates *q* of a beam node which captures the deformation of its cross section are defined by a positional vector $x\in R^{3}$ and a rotation matrix **R** ∈ *SO*(3). Its general velocities $\dot{q}$ are defined using a linear velocity vector $v\in R^{3}$ and an angular velocity vector $\omega \in R^{3}$ . We use the following update rule to integrate the new positions and orientations of the beam cross section from the end of time step linear and angular displacements:

$$\begin{array}{cc} x_{+} & =x+hv_{+}\\ R_{+} & =\exp\left(h{\left[\omega_{+}\right]}_{\times }\right)R \end{array}$$ (6)

With [⋅]_×_ the operator which converts a vector into a skew symmetric matrix

$${\left[a\right]}_{\times }=\left(\begin{array}{ccc} 0 & -a_{3} & -a_{2}\\ a_{3} & 0 & -a_{1}\\ a_{2} & a_{1} & 0 \end{array}\right)$$

And exp the matrix exponential function whose closed form in the case of skew symmetric matrices is given by the Euler Rodrigues formula.

**FIGURE 1:**
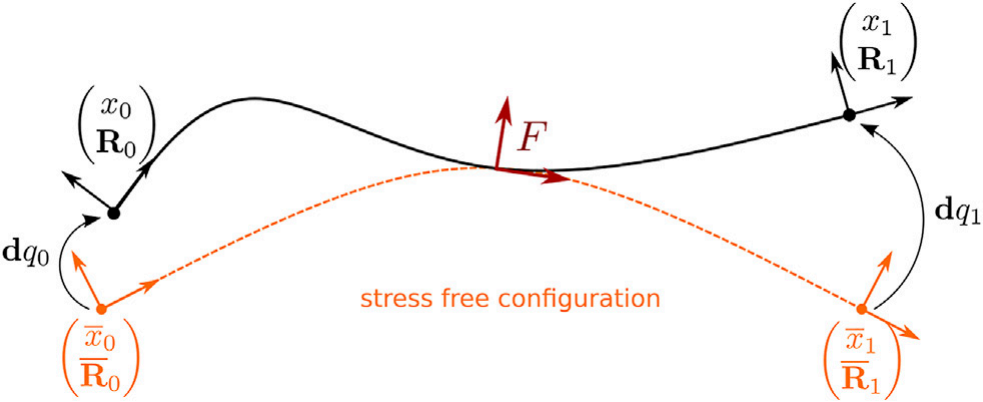
The computation of the deformation is done in the local reference frame *F* of the beam element (in red). Using this frame, the 6D local displacements of the nodes are computed between the current configuration and their rest configuration (in orange).

**FIGURE 2:**
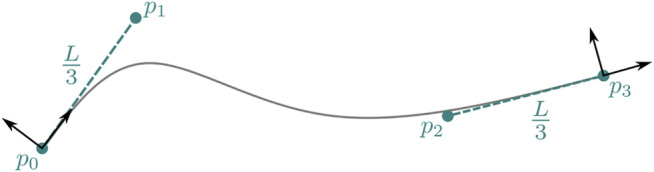
Construction of the spline control points from the beam end nodes.

Using the corotational method, we decompose the motion of a flexible beam in two parts, a rigid body motion and a deformation motion. We add, to each flexible beam, a reference frame *F* = [*x*_*F*_, **R**_*F*_] at the center of the element and we use it as a local coordinate system where the deformation is measured (see Figure 1). To define the orientation of this local frame *F*, we first construct a cubic spline using the position and orientation of the beam end nodes. The position of the control points at both end of the cubic spline is given by the position of the corresponding beam nodes. The second and third control points are constructed using the orientation of the **u**_*x*_ axis of each node and the rest length *L* of the beam (see Figure 2).

$$p_{0}=x_{0}\;p_{1}=x_{0}+\frac{L}{3}R_{0}\left(\begin{array}{c} 1\\ 0\\ 0 \end{array}\right)\;p_{2}=x_{1}-\frac{L}{3}R_{1}\left(\begin{array}{c} 1\\ 0\\ 0 \end{array}\right)\;p_{3}=x_{1}$$ (7)

From the control points we can define the position *x*_*F*_ of corotational frame *F* at the mid point of the beam element, by evaluating the interpolation function of the cubic spline at the parameter value 0.5.

$$p\left(u\right)={\left(1-u\right)}^{3}p_{0}+3u{\left(1-u\right)}^{2}p_{1}+3u^{2}\left(1-u\right)p_{2}+u^{3}p_{3}$$ (8)

**FIGURE 3:**
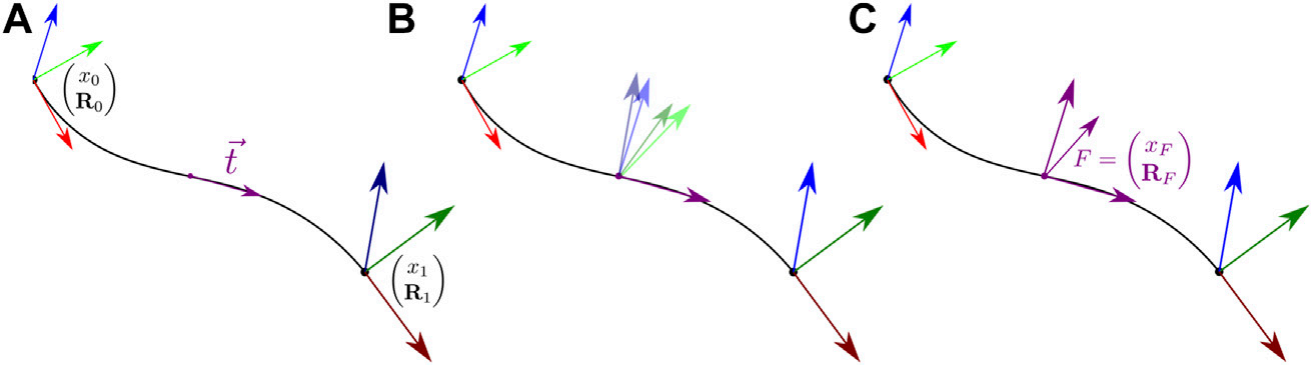
To construct **R**_*F*_ we first evaluate the tangent at the cubic Bézier curve mid point **(A)** We then transform each beam end point orientation to align its first axis with the mid point tangent **(B)** We finally compute the orientation of *F* by taking the average of these two rotations **(C)**.

The steps used to construct the orientation **R**_*F*_ of corotational frame *F* are illustrated in Figure 3.

First, we evaluate the mid point tangent $\vec{t}$ by taking the normalized gradient of the cubic spline interpolation function.

$$\nabla p\left(u\right)={\left(1-u\right)}^{2}3\left(p_{1}-p_{0}\right)+2{\left(1-u\right)}^{2}3\left(p_{2}-p_{1}\right)+u^{2}3\left(p_{3}-p_{2}\right)$$ (9)

$$\vec{t}=\frac{\nabla p\left(0.5\right)}{\left\|\nabla p\left(0.5\right)\right\|}$$

**FIGURE 4:**
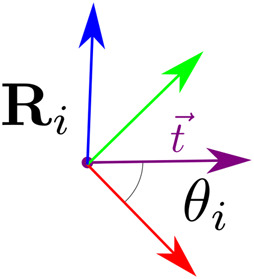
Alignment of $u_{x_{i}}$ with the mid point tangent.

Then, for each beam end node we find the rotation ***R'***_*i*_ which aligns the node $u_{x_{i}}$ axis with the mid point tangent $\vec{t}$ (see Figure 4). From there, the orientation of *F* is derived with spherical linear interpolation.

We then construct **R**_*F*_ using:

$$\begin{array}{ll} {R^{'}}_{i} & =R_{\theta_{i}}R_{i}\\ R_{F} & ={R^{'}}_{0}\exp\left(0.5\cdot \log\left({R^{'}}_{1}^{⊤}{R^{'}}_{0}\right)\right) \end{array}$$

#### 3.2.2 Elastic Force and Stiffness

The assumption of the corotational method is that in the reference frame, the deformation remains small at the level of each element, and we can compute in the local frame the element force **f**_**e**_ with the linear relation:

$$f_{e}=K_{e}\left(u-\bar{u}\right)$$ (10)

With.

• $u=F^{⊤}\left[\begin{array}{c} q_{0}\\ q_{1} \end{array}\right]$ the column vector containing the coordinates of the beam nodes in the deformed configuration expressed in its local frame *F*.
• $\bar{u}={\bar{F}}^{⊤}\left[\begin{array}{c} {\bar{q}}_{0}\\ {\bar{q}}_{1} \end{array}\right]$ the column vector containing the coordinates of the beam nodes in the stress free configuration expressed in its local frame $\bar{F}$ .
• **K**_**e**_ the 12 × 12 elementary stiffness matrix given by Przemieniecki (1985).

The elementary stiffness matrix **K**_**e**_ relates the positional and rotational motion of each of the beam nodes to forces and torques applied to them.

With $G=\frac{E}{2*\left(1+\nu \right)}$ where *E* is the Young modulus and *ν* Poisson ratio; *A* is the cross-sectional area of the beam, *l* is its length; *I*_*y*_ and *I*_*z*_ are the cross-section moments of inertia; Φ_*y*_ and Φ_*z*_ are deformation parameters related to the shearing of the cross section and are given by the following relation: $\Phi_{y}=\frac{12EI_{z}}{GA_{sy}l^{2}}$ and $\Phi_{z}=\frac{12EI_{y}}{GA_{sz}l^{2}}$ with *A*_*sy*_ and *A*_*sz*_ the shearing area in the *y* and *z*.

We use the orientation of corotational frame **F** to transform the force **f** = **R**_*F*_ **f**_**e**_ and the stiffness matrix $K=R_{F}K_{e}R_{F}^{⊤}$ from the local to the global coordinate system.

### 3.3 Length-Preserving Lagrangian Constraint

To model a suture thread, we need to equip the beam model with an additional length-preserving constraint. Indeed, during suturing, the traction loads applied to the thread are very high, and a simple increase of the beam rigidity in the stretching direction cannot enforce the inextensibility property.

For a Bézier curve, we can compute its length *L* with the arc length function

$$L=\int_{0}^{1}\|\nabla p\left(u\right)\|\;du$$ (11)

In the case of cubic Bézier curves this function has no closed form solution, and we use Gauss quadrature to approximate the integral. We sample the integral using the following Gauss points (with $A=2\sqrt{6/5}$ )

$$\begin{array}{llll} u_{0} & =\frac{-\sqrt{\left(3-A\right)/7}+1}{2} & u_{1} & =\frac{\sqrt{\left(3-A\right)/7}+1}{2}\\ u_{2} & =\frac{-\sqrt{\left(3+A\right)/7}+1}{2} & u_{3} & =\frac{\sqrt{\left(3+A\right)/7}+1}{2} \end{array}$$

Which using the quadrature rule gives

$$L\approx \frac{18+\sqrt{30}}{72}\left(\left\|\nabla p\left(u_{0}\right)\|+\|\nabla p\left(u_{1}\right)\right\|\right)+\frac{18-\sqrt{30}}{72}\left(\left\|\nabla p\left(u_{2}\right)\|+\|\nabla p\left(u_{3}\right)\right\|\right)$$ (12)

For each beam segment, we can then formulate the length-preserving constraint equation as a composition of two functions of the beam generalized degrees of freedom

$$\Phi \left(q\right)=\Phi_{2}◦\Phi_{1}\left(q\right)-\bar{L}$$ (13)

Where.

• Φ_1_ is the function which gives the position of the control points **p** in terms of the beam end nodes $q=\left(\begin{array}{c} q_{1}\\ q_{2} \end{array}\right)$ . See Eq. 7
• Φ_2_ the function which gives the length of the cubic Bézier curve *L* in terms of its four control points **p**_0_ … **p**_3_. See Eq. 11
• $\bar{L}$ is the length of the beam segment in its stress free configuration, $\bar{L}=\Phi_{2}◦\Phi_{1}\left(\bar{q}\right)$

Linearizing Φ with respect to the generalized coordinates of the beam gives the constraint Jacobian **H**, which in this case is a 1 × 12 matrix

$$\begin{array}{ll} \frac{\partial \Phi }{\partial q} & =H=\frac{\partial \Phi_{2}}{\partial p}\cdot \frac{\partial \Phi_{1}}{\partial q}\\ \frac{\partial \Phi_{1}}{\partial q} & =H_{1}=\left(\begin{array}{cccc} I_{3} & 0 & 0 & 0\\ I_{3} & -{\left[p_{1}-p_{0}\right]}_{\times } & 0 & 0\\ 0 & 0 & I_{3} & -{\left[p_{2}-p_{3}\right]}_{\times }\\ 0 & 0 & I_{3} & 0 \end{array}\right)\\ \frac{\partial \Phi_{2}}{\partial p} & =H_{2}=\left(\begin{array}{cccc} \int_{0}^{1}\frac{\partial \|\nabla p\left(u\right)\|}{\partial p_{0}}\;du & \int_{0}^{1}\frac{\partial \|\nabla p\left(u\right)\|}{\partial p_{1}}\;du & \int_{0}^{1}\frac{\partial \|\nabla p\left(u\right)\|}{\partial p_{2}}\;du & \int_{0}^{1}\frac{\partial \|\nabla p\left(u\right)\|}{\partial p_{3}}\;du \end{array}\right) \end{array}$$

Again, since no close form for the integrals which compose the components of $\frac{\partial \Phi_{2}}{\partial p}$ exists, we also resort to Gauss quadrature to evaluate the terms numerically.

Note that Φ is highly non-linear, so depending on the chosen time integration and numerical resolution solver, it may be necessary to apply an adequate stabilization strategy.

### 3.4 Contact and Collision Model of the Suture Thread

To be able to perform knots we need to robustly capture thread-thread interactions. In our simulation we model contact using the Signorini’s contact law (Duriez et al., 2006) which gives a complementary condition between the contact force *λ* and the proximity distance (or gap) function **g**(*q*)

0≤λ⊥g(q)≥0 (14)

We use collision detection to discretize the distance function **g** for the suture thread by computing at each time step its closest vertex-edge and edge-edge features. We use bounding volume hierarchies (BVH) to prune distant features to focus the computations on features that are close by (see Bergen (1997) for instance).

It is common in simulators to model the geometry of the suture thread with piecewise continuous geometry like line segments (Wang et al., 2017), (Qi et al., 2017), or cylinders with fixed radius (Hüsken et al., 2013). Because each geometry primitive is only locally continuous and not smooth, discretization artifacts will always occur. In our work, we instead rely on the cubic Bézier geometry associated with each beam segment to get an accurate and spatially continuous contact definition.

#### 3.4.1 Vertex-Edge Distance Function

The closest point on a cubic Bézier curve from another curve vertex $q\in R^{3}$ can be obtained by minimizing the following square distance function

$$d\left(u\right)=\frac{1}{2}\left(p\left(u\right)-q\right)\cdot \left(p\left(u\right)-q\right)$$

A minimum *u*_*min*_ of the **d** function is reached when its gradient is zero

∇d(u)=∇p(u)⋅(p(u)−q)

Since no closed form exists to express the minimum of the ∇**d** function we use a Newton Raphson iterative scheme to converge toward a local minimum of the function. This requires at each iteration the evaluation of the Hessian *H*(**d**) of the square distance function which is given by

H(d)(u)=H(p)(u)⋅(p(u)−q)+∇p(u)⋅∇p(u)

#### 3.4.2 Edge-Edge Distance Function

The closest points between two cubic Bézier curves **p** and **q** with parameters *u* and *v* can be obtained by minimizing the square distance function

$$d\left(u,v\right)=\frac{1}{2}\left(p\left(u\right)-q\left(v\right)\right)\cdot \left(p\left(u\right)-q\left(v\right)\right)$$

Again, to find a local minimum (*u*_*min*_, *v*_*min*_) of this function, we look for a root of its the gradient

$$\nabla d\left(u,v\right)=\left(\begin{array}{c} \nabla p\left(u\right)\cdot \left(p\left(u\right)-q\left(v\right)\right)\\ -\nabla q\left(v\right)\cdot \left(p\left(u\right)-q\left(v\right)\right) \end{array}\right)$$

The Hessian used during each Newton Raphson iteration being

$$H\left(d\right)\left(u,v\right)=\left(\begin{array}{cc} H\left(p\right)\left(u\right)\cdot \left(p\left(u\right)-q\left(v\right)\right)+\nabla p\left(u\right)\cdot \nabla p\left(u\right) & -\nabla p\left(u\right)\cdot \nabla q\left(v\right)\\ -\nabla p\left(u\right)\cdot \nabla q\left(v\right) & H\left(q\right)\left(v\right)\cdot \left(q\left(v\right)-p\left(u\right)\right)+\nabla q\left(v\right)\cdot \nabla q\left(v\right) \end{array}\right)$$

#### 3.4.3 Collision Detection

To produce the contact geometry, we run collision detection at every time step of the simulation. Collision detection typically uses two stages to produce the contact information. The first stage, called the broad phase algorithm, uses bounding volume hierarchies—a hierarchy of axis aligned bounding boxes in our simulation—to prune distant features. The only cubic Bézier lines that are tested for proximity are the one which are enclosed by intersecting bounding boxes. The second stage, called the narrow phase, uses the potential colliding pair of primitives provided by the broad phase to test for potential contact by computing the Vertex-Edge and Edge-Edge distance. The result of the narrow phase gives us the contact geometry, from which we can generate non-penetration constraints.

#### 3.4.4 Unilateral Contact Constraint

Sampling the distance function using closest point features gives us a list of pairs of contact points together with their parametric location on the cubic Bézier curve and their contact normal. For each pair (**p**(*u*), **q**(*v*)) of contact points the non-penetration contact constraint can be written as

$$n^{⊤}\left(p\left(u\right)-q\left(v\right)\right)-2r-\epsilon \geq 0$$ (15)

With.

• $n=\frac{p\left(u\right)-q\left(v\right)}{\left\|p\left(u\right)-q\left(v\right)\right\|}$ the contact normal
• *r* the radius of the suture thread
• *ϵ* is a user-defined safety tolerance

To enforce the non-penetration constraint at the end of the time step we linearize the gap function with respect to the degrees of freedom *q*

$$g\left(q^{+}\right)\approx g\left(q\right)+\frac{\partial g}{\partial q}\left(q^{+}-q\right)$$ (16)

The constraint Jacobian $H_{n}=\frac{\partial g}{\partial q}$ at a contact location **p**(*u*) with normal **n** being

$$\begin{array}{ll} H_{n} & =n\cdot \left(\begin{array}{c} \frac{\partial p\left(u\right)}{\partial p_{i}} \end{array}\right)\cdot \left(\begin{array}{c} \frac{\partial p_{i}}{\partial q_{j}} \end{array}\right)\\ H_{n} & =n\left(\begin{array}{cccc} {\left(1-u\right)}^{3} & u{\left(1-u\right)}^{2} & u^{2}\left(1-u\right) & u^{3} \end{array}\right)\left(\begin{array}{cccc} I_{3} & 0 & 0 & 0\\ I_{3} & -{\left[p_{1}-p_{0}\right]}_{\times } & 0 & 0\\ 0 & 0 & I_{3} & -{\left[p_{2}-p_{3}\right]}_{\times }\\ 0 & 0 & 0 & I_{3} \end{array}\right) \end{array}$$

We can then express the contact constraint inequality at the velocity level as.

$$\begin{array}{ll} A\Delta \dot{q} & =H_{n}^{⊤}\lambda_{+} \end{array}$$ (17)

$$\begin{array}{ll} \Delta \dot{q} & ={\dot{q}}_{+}-{\dot{q}}_{*} \end{array}$$ (18)

$$\begin{array}{ll} 0\leq \lambda_{+} & ⊥H_{n}\Delta \dot{q}\geq -\frac{1}{h}g\left(q\right)-H_{n}{\dot{q}}_{*} \end{array}$$ (19)

#### 3.4.5 Coulomb Frictional Contact

Friction plays an important role during the formation of knots, where the suture thread motion alternates between sticking and sliding. For each contact, we construct an orthonormal frame **n**, **t**_1_, **t**_2_ to measure the tangential gap together with the normal gap.

Noting *μ* the friction coefficient, the Coulomb’s law can be then written as.

$$\begin{array}{ll} A\Delta \dot{q} & =H_{n}^{⊤}\lambda_{n_{+}}+H_{t}^{⊤}\lambda_{t_{+}} \end{array}$$ (20)

$$\begin{array}{ll} 0\leq \lambda_{n_{+}} & ⊥H_{n}\Delta \dot{q}\geq -\frac{1}{h}g\left(q\right)-H_{n}{\dot{q}}_{*} \end{array}$$ (21)

$$\begin{array}{ll} \|\lambda_{t_{+}}\|\leq \mu \lambda_{n_{+}} & ⊥H_{t}\Delta \dot{q}\geq -H_{t}{\dot{q}}_{*} \end{array}$$ (22)

### 3.5 Suture Thread Tension

At each time step of the simulation the solution of the reduced system gives us the intensity $\lambda_{l_{+}}$ of the forces that need to be applied to preserve the length of each of the beam segments that compose the suture thread deformable model. These intensities directly reflect the degree of tension on the suture thread, and we use them to construct the colour code to provide visual haptic cues. For each type of suture thread we specified a rupture threshold *λ*_*yield*_ above which breakage occurs, and defined a colour code accordingly to notify the user.

The mechanical work $\left\|h\lambda_{l_{+}}\left(-\frac{1}{h}\Phi_{l}\left(q\right)-H_{l}^{⊤}{\dot{q}}^{*}\right)\right\|$ is the energy that needs to be injected in the system to respect the length-preserving constraint during the time step, and directly depends on the mechanical parameters of the suture thread.

**FIGURE 5:**
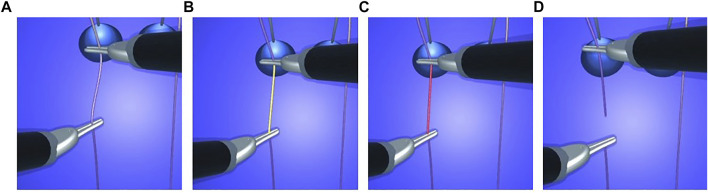
Visual stress on a thread from floppy **(A)** to tight **(B,C)** condition until suture breakage with tension release **(D)**.

Based on this energy, we render a gradient of signal colours (from yellow to red) on the stretched areas of the suture thread (see Figures 5, 13). When the thread is subject to excessive force, it turns red until reaching the breakage point, after which it switches back to its normal colour without any tension (Figure 5).

### 3.6 Summary

We use a continuous representation of the suture thread based on cubic Bézier curves to address the stability challenges that arise during the formation of knots in the simulation. From this continuous setting, we obtain an accurate definition of the suture thread length from which a length-preserving constraint function can be derived. Additionally, as the support geometry is continuous and smooth, we generate spatially continuous contact geometry which can capture precisely the moment at which a knot is formed in the simulation. The intensity of the length-preserving constraint can be mapped to a colour code reflecting the proficiency of the user manipulation.

## 4 Results

Standard robotic knot-tying and suturing tasks were performed on a current robotic surgical system (da Vinci Xi, Intuitive Surgical) in order to assess comparability with the proposed simulation model. Both the robotic and the simulated knot-tying and suturing tasks were recorded on video for visual comparison of suture thread behavior during manipulation. The Fundamentals of Robotic Surgery (FRS) dome was chosen as a benchmark model for robotic surgical skills training Satava et al. (2019). Measures were taken of the FRS dome and of commonly used suture threads for an accurate representation in the robotic simulation module.

The simulations were computed in real time at over 50 frames per second on a computer with an AMD Ryzen 9 5900X CPU, a NVIDIA GeForce RTX 3070 GPU, and 64 GB of RAM. As the simulation scenarios are sufficiently simple, there was no need for specific efforts to reach this 50 frames per second target. For integration of more complex simulations, we envision several ways to enhance the computational performances of our simulation if needed, such as work by Otaduy et al. (2009) and Hecht et al. (2012).

The results presented are subdivided into the following categories:

• A series of behavior tests to illustrate the soundness of our model (Section 4.1)
• The suture thread model properties and its applications to knot-tying and suturing (Section 4.2)
• The visual haptic feedback (Section 4.3)
• The comparison of the knot-tying exercise between the simulation and the reality on a da Vinci Xi system (Section 4.4)

### 4.1 Thread Behavior Tests

**FIGURE 6:**
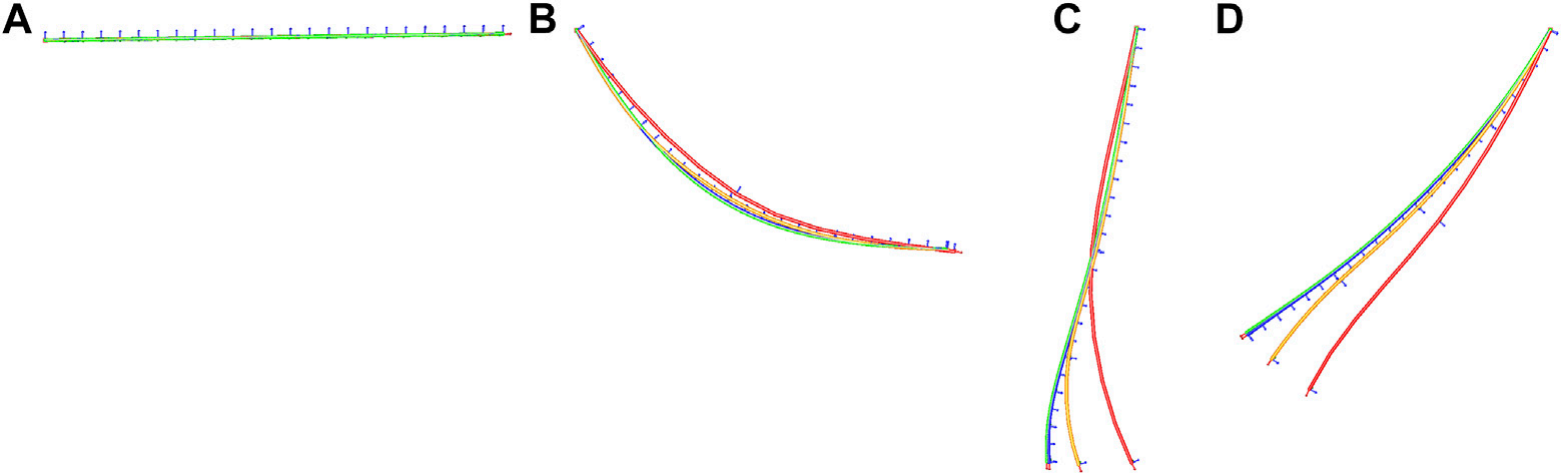
We test four different discretizations of a suture thread of 12 cm subjected to gravity. In increasing order of discretization red—orange—blue—green. The dynamics of the pendular motion is captured at time 0[*s*] **(A)**, 0.1[*s*] **(B)**, 0.2[*s*] **(C)** and 0.3[*s*] **(D)**. The model converges to a unique solution as the level of discretization increases.

**FIGURE 7:**
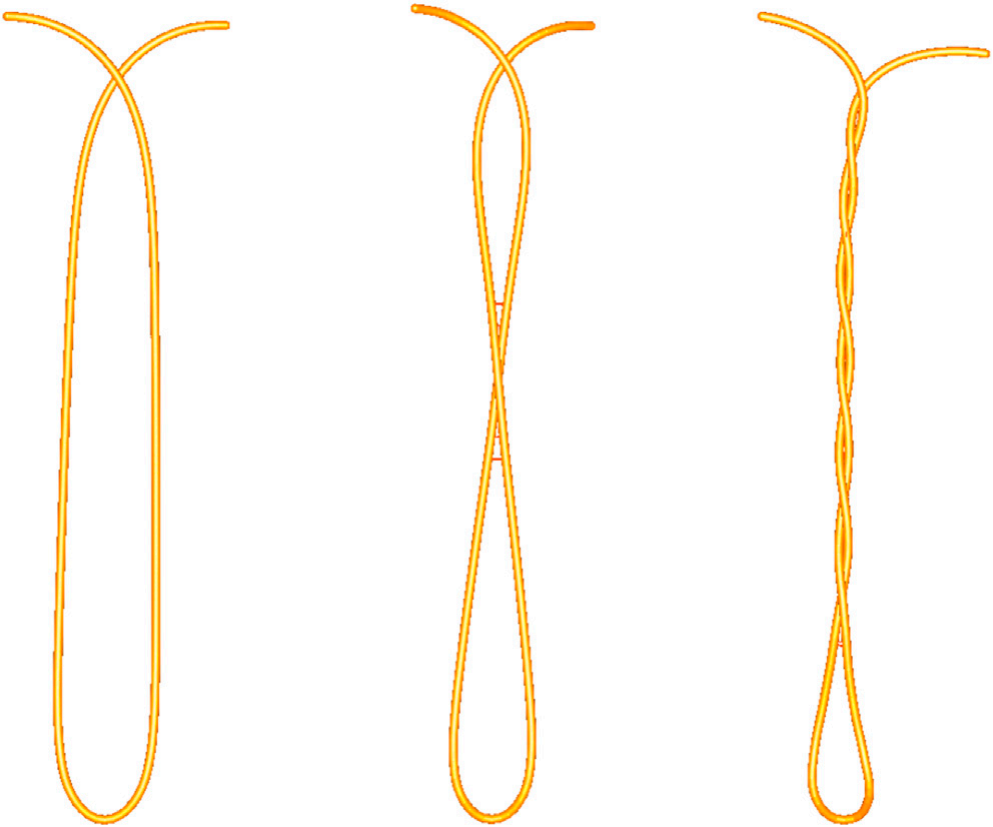
We apply a torsional motion at the end points of the thread. The coupling between bending and torsion allows the formation of plectonemes.

**FIGURE 8:**
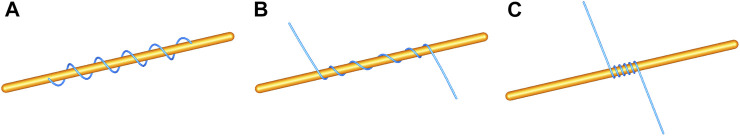
A suture thread is wrapped around a fixed rigid cylinder by pulling at its ends. The wrapping motion is sampled at time 0 [*s*]**(A)**, 0.5 [*s*]**(B)**, and 1 [*s*]**(C)**. The model remains inextensible during this simulation, which is why the loops around the rigid cylinder get closer and closer.

**FIGURE 9:**
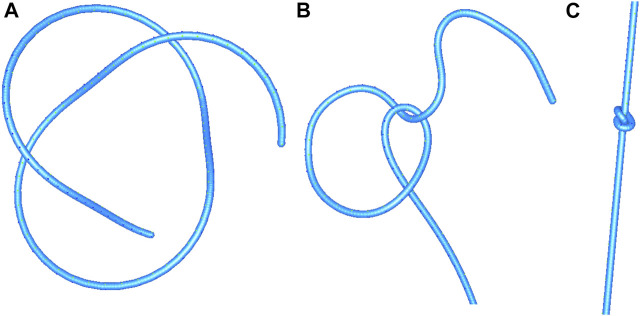
Gradual tightening of a simple knot. The motion is sampled at time 0 [*s*]**(*A*)**, 1 [*s*]**(*B*)**, and 2 [*s*]**(*C*)**. The accurate contact model allows to form the knot and to tighten it.

In order to test the behavior of our model we reproduced several of the experiments proposed in Xu and Liu (2018). These interactive experiments helped to confirm that our deformation model converges to a unique solution as the mesh discretization increases (Figure 6), its ability to reproduce the well known phenomena of plectonemes (Figure 7), its ability to remain inextensible even in scenarios where the suture thread is highly constrained (Figure 8), and finally its ability to form a knot (Figure 9).

### 4.2 Suture Thread Model

#### 4.2.1 Mechanical Properties

We developed the deformation model of the suture thread proposed in Section 3.2. The model reproduces a realistic thread behaviour during manipulation, which was demonstrated in comparison with several types of surgical suture threads. Figure 7 illustrates the coupling between torsion and bending when applying a torsional load at both ends of the thread. In order to preserve movement dynamics for a realistic gesture, the model is damped at a low level (the minimum needed for the purpose of numerical stability). In contrast to models encountered in common robotic VR simulations, our model overcomes the challenge of thread inextensibility (see Section 3.3). The strategy is sufficiently robust to offer an immersive thread manipulation to the user.

#### 4.2.2 Collision

The developed method (see Section 3.4) to handle thread contact is robust enough to support various manipulations including thread wrapping around instruments, thread-thread interaction, as well as contact between the thread and other elements. During the knot-tying task, the thread touches the rings (linear collision model) and the dome (triangular collision model). It is in contact with the instruments when being grasped, and when being wrapped around an instrument tip for single or multiple loops. When the knot is formed, but not yet pulled tight, the thread is mainly in self-collision. We introduce an offset between the contacts to prevent the suture thread from tunneling (typically a small fraction of the thread’s radius, see *ϵ* parameter in Eq. 15). Maintaining a minimal distance between adjacent threads enables precise recognition of the created pattern. If the pattern does not correspond to the desired one, it can thus be identified and corrected.

#### 4.2.3 Knot Tying

**FIGURE 10:**
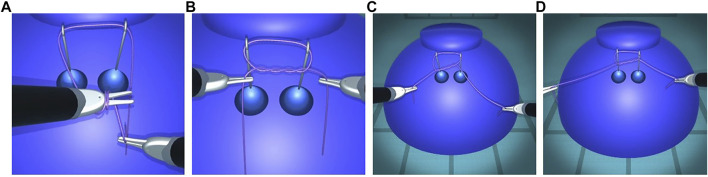
First step of a surgeon’s knot. The double overhand knot is created by wrapping the thread around an instrument tip **(A)**, grasping the other end, and pulling it through the double loop, resulting in a pattern with two twists **(B)**. The knot position can be adjusted by exerting traction on one end, thus moving the knot over to the other end **(C,D)**. The collision is sufficiently robust to support thread interactions with itself and with the other elements.

The present method enables the user to tie various knots in different scenarios and correct them if needed. As an example, the first step of the blocking sequence of a surgeon’s knot is a double overhand knot. It is a half knot with an extra turn, illustrated in Figure 10. The double loop provides additional friction to impede loosening of the first while the subsequent throws are performed. Before tightening, the knot position can be adjusted by means of traction on one end of the thread while holding the other end loosely. This way, the knot moves over to the desired position and the remaining length of each end can be optimized for subsequent knots (Figure 10C,D). The surgeon’s knot is then completed by adding a half knot wrapped in the opposite sense, and additional knots can be added (sequence in Figure 15).

**FIGURE 11:**
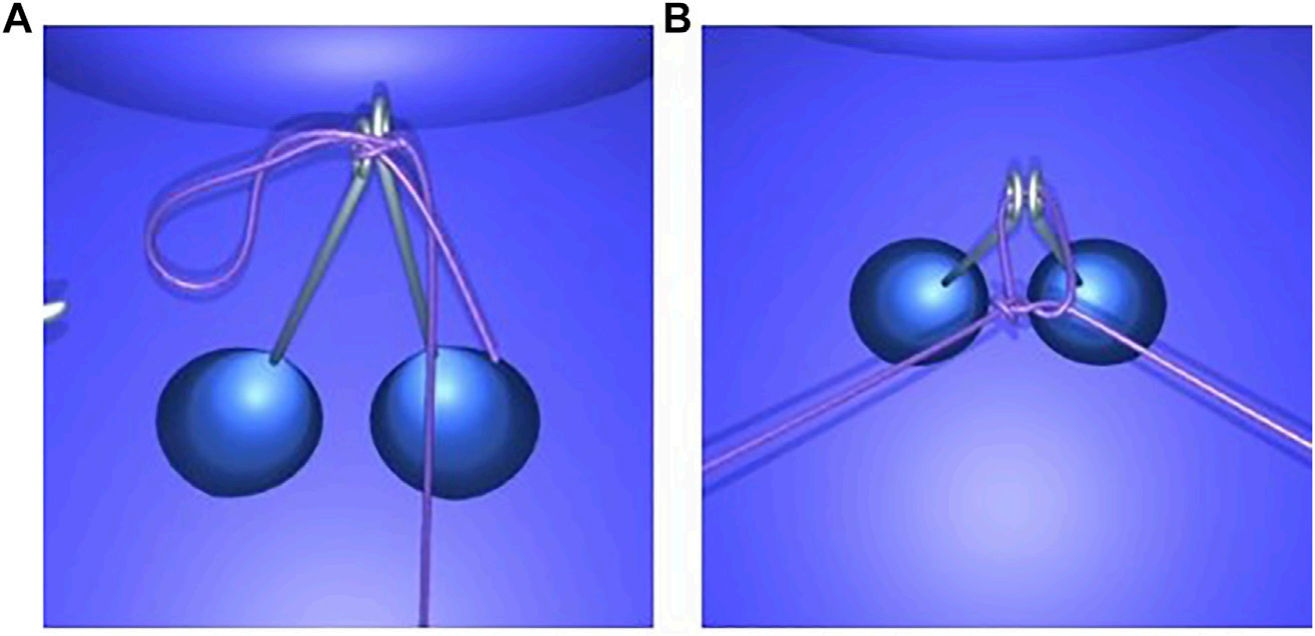
Simulation accurately reproducing knot-tying errors. An accidental loop is created when one end of the thread is not completely pulled through the loop before tightening the knot on the rings **(A)**. An air knot is created when the knot is not properly guided down onto the rings before tightening it with traction on both ends of the thread **(B)**.

In contrast to other existing simulators, our approach does not presuppose the knot pattern by use of an analysis of the tool gesture. In the present approach, the precise mechanics and collision lead to the exact patterns that correspond to the performed manipulation. Consequently, our simulation approach complies with errors due to incorrect loop formation or knot tightening. This results in an accurate representation of the faulty pattern, just like with a real thread (see Figure 11). These precise thread characteristics are key to allow to substitute a robotic knot-tying and suturing training on the real console system with a simulation module.

#### 4.2.4 Suturing

**FIGURE 12:**
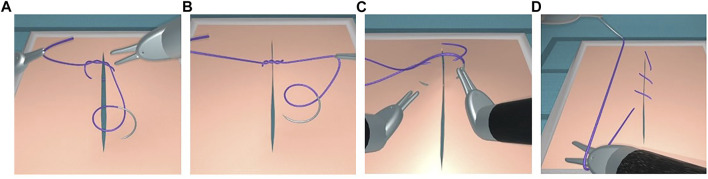
Interrupted suturing **(A,B)** and continuous suturing **(C,D)**.

Based on the proposed approach, we developed interrupted suturing and continuous suturing tasks (see Figure 12). In these modules, the thread is attached to a needle. The collision strategy of the rigid needle is the same as the one described for the thread. The interrupted suturing task involves placing the stitch and surgical knot-tying as outlined above. For the continuous suturing task, one end of the thread is anchored at the edge of the incision. The suture can be gradually tightened during each step of the task, and can be corrected at the end in order to ensure that the gap is well closed. Finally, a pair of scissors (third instrument) allows to cut the thread at the end of each task.

### 4.3 Visual Haptic Feedback

In order to reproduce the conditions of the majority of current robotic surgical systems, our simulation does not provide haptic feedback. Instead, we augment the graphics rendering of the simulation with visual cues. Visual force feedback has been demonstrated to enhance knot quality and reduce applied forces as well as suture breakage rates in robotic suturing Reiley et al. (2008). By mapping the intensity of the length-preserving constraints in a colour-code, our model indicates the degree of closeness to the rupture threshold of a suture thread or to damage from shear forces on tissues.

Such visual cues alert users that errors are about to occur when continuing to work with excessive force. In order to demonstrate the consequences of inappropriate handling, our simulation still progresses at this point and includes suture breakage or tissue tearing conditions (see Figure 5). This section presents the different types of visual cues we propose in our simulator.

#### 4.3.1 Stress on Tissue

**FIGURE 13:**
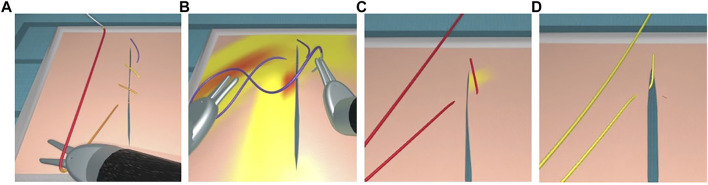
Visual stress gradients on a thread when tightening a continuous suture **(A)**, and on tissue when driving a needle through tissue (right instrument) while applying a counter-pressure (left instrument) **(B)**. Combined visual cues on thread and tissue when applying excessive force **(C)** leading to a tear in the tissue and subsequently reduced tension on the thread **(D)**.

Our simulator represents stress on tissue with the same colour code. The signal colours appear on the stressed area, such as when puncturing with a needle or when applying counter-pressure with the needle driver (Figure 13B). Moreover, when excessive traction is applied, tissue tearing can occur, as represented with a red mark on the tissue where the thread has torn through (Figure 13 C, D).

#### 4.3.2 Stress on Rigid Objects

**FIGURE 14:**
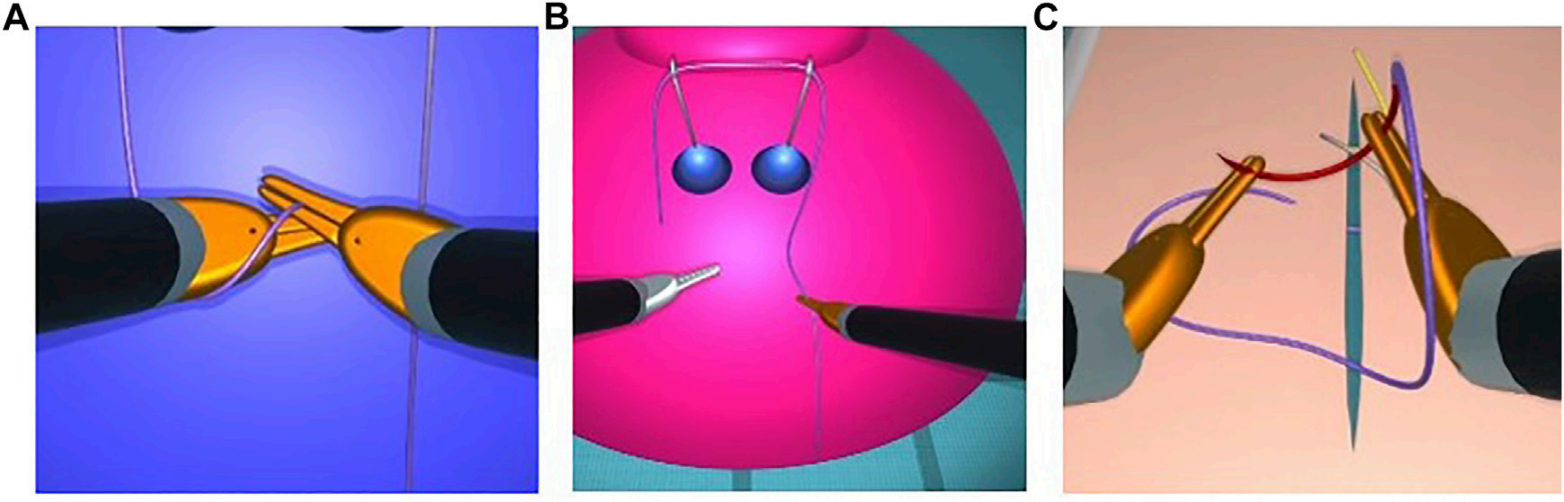
Visual cues for excessive forces during collisions: Visual cues for excessive forces during collisions. Instrument tips turning from silver to orange during collision when forming a loop with a thread for knot-tying **(A)**, right instrument tip turning orange during collision with the dome, which turns from blue to pink **(B)**, double grasping with shearing forces during needle manipulation with both instrument tips turning orange, and the needle approximating the breakage point as represented by its color change to red **(C)**. Animated scenes are shown in the Supplementary Video S1.

When rigid objects are subject to excessive force, a change in color alerts the user of a collision (see Figure 14). If the collision is resolved, these objects return to their original colour. In contrast, without a trajectory correction the simulation can become unstable and a game-over state is reached.

### 4.4 Preliminary Evaluation by Surgeons

**FIGURE 15:**
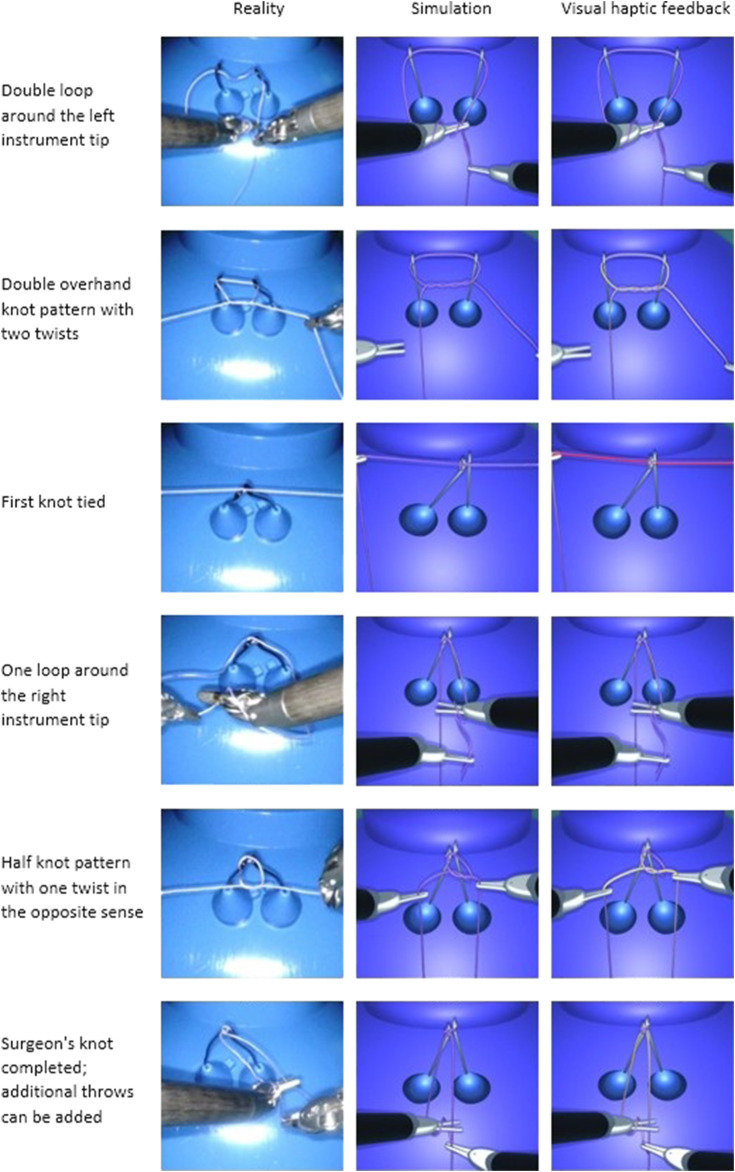
Task performance comparison between a real robotic system and the simulation. The knot-tying sequence is based on a surgeon’s knot. The task with the da Vinci Xi system is shown on the left, and the simulated task is shown without (middle) and with visual haptic cues (right).

At the IRCAD Strasbourg training platform, a trained robotic surgeon (BS) performed and recorded various knot-tying and suturing steps using the da Vinci Xi surgical system (Intuitive Surgical). The same tasks were then performed on the simulator by several of the authors. The gestures performed were slow on purpose, in order to ensure visibility of the thread behaviour and knot pattern in the video recordings. According to the measures taken on the real FRS dome, a stylized FRS dome version was implemented into the simulation module for the task of knot-tying around rings known from the FRS curriculum (see https://frsurgery.org/). During development of the simulation approach, instrument articulation and range were adjusted to represent the typical degrees of freedom and thread movements observed in the real scenario. The FLS curriculum (required for US board certification in general surgery) includes suturing with intracorporeal knot-tying as one of the five laparoscopic tasks. After placing a stitch, it comprises a sequence of knots: a double overhand knot followed by two half knots (Qi et al., 2017). Consequently, this step sequence was chosen to illustrate the degree of realism in Figure 15. The suturing and knot-tying simulation exercises were then demonstrated to various surgeons. During the French national surgical conference 2021 (Association Française de Chirurgie, AFC), feedback was collected from 20 surgeons. They all underlined the plausibility of the thread simulation. The main positive feedback aspects were the overall realism of the suture, its fluidity, and the precise control of the knot tightening. The visual cues, and in particular the visual stress on suture threads, were perceived as a valuable tool to prevent beginners from breaking the suture. As a current limitation, our thread simulation lacks performance metrics (position, instrument path, duration, etc.). These will further be implemented in order to perform a study quantitatively assessing trainee performance with and without visual haptic cues, and evaluate training progress in comparison with commercial simulation systems.

For a braided absorbable suture (Vicryl 3–0, Ethicon) the characteristics compared to the simulated one are as follows:

**Table:** 

Parameters	Reality	Simulation
Young Modulus	7 GPa	3 GPa
Poisson ratio	0.35	0.35
Diameter	0.25 mm	1.5 mm/0.4 mm (*)
Mass density	1.530 g.cm-3	1.530 g.cm-3
Length	165 mm	165 mm

(*) 1.5 mm for mechanics and 0.4 mm for collision.

For numerical stability purposes, we increased the mechanical diameter of our model, and then reduced the Young Modulus to compensate.

## 5 Conclusion

We present novel contributions on the wire model with constraints on elongation and on the continuous modeling of the contacts. The robust collision detection and suture thread modeling enable suturing and knot-tying in an interactive and real-time simulation environment running on a standard personal computer. Based on these advancements, virtually all types of thread manipulation can be performed in the simulation, just as in real life, and the knot can be tightened relatively far without compromising stability. Our simulation integrating visual haptic cues has the potential to facilitate learning of suturing and knot-tying tasks, since visual haptic feedback was already shown to be advantageous for novice surgeons to reduce applied forces and avoid suture breakage. Moreover, numerous interactive tests are provided to assess the quality of the results. Additionally, we compare the simulation results with a suture task performed with a da Vinci robotic system. Future work will include a model of the wire plasticity. Additionally, assessment metrics such as task completion time, accomplishment of the required knot-tying sequence and knot tightness will be integrated. Based on physical forces/stresses calculated in the simulation, exerted forces during a task can be extracted and used as performance metrics. Comparison of learning curves with and without visual haptic cues will then quantify the economy of motion and presumed learning advantage for training with visual haptic feedback. Performance evaluation scores with quantitative data will enable objective training assessment and inter-user comparability.

## Data Availability

The original contributions presented in the study are included in the article/Supplementary Material, further inquiries can be directed to the corresponding author.
